# Europinidin Inhibits Rotenone-Activated Parkinson’s Disease in Rodents by Decreasing Lipid Peroxidation and Inflammatory Cytokines Pathways

**DOI:** 10.3390/molecules27217159

**Published:** 2022-10-23

**Authors:** Ali Altharawi, Khalid M. Alharthy, Hassan N. Althurwi, Faisal F. Albaqami, Sami I. Alzarea, Fahad A. Al-Abbasi, Muhammad Shahid Nadeem, Imran Kazmi

**Affiliations:** 1 Department of Pharmaceutical Chemistry, College of Pharmacy, Prince Sattam Bin Abdulaziz University, Al-Kharj 11942, Saudi Arabia; 2 Department of Pharmacology and Toxicology, College of Pharmacy, Prince Sattam Bin Abdulaziz University, Al-Kharj 11942, Saudi Arabia; 3 Department of Pharmacology, College of Pharmacy, Jouf University, Aljouf, Sakaka 72341, Saudi Arabia; 4 Department of Biochemistry, Faculty of Science, King Abdulaziz University, Jeddah 21589, Saudi Arabia

**Keywords:** europinidin, oxidative stress, Parkinson’s disease, rotenone

## Abstract

Background: Europinidin is a derivative of delphinidin obtained from the plants *Plumbago Europea* and *Ceratostigma plumbaginoides*. This herb has wide medicinal applications in treating various diseases but there are very few studies available on this bioactive compound. Considering this background, the present study is designed for the evaluation of Europinidin against Parkinson’s disease. Aim: The investigation aims to assess the effect of Europinidin in the rotenone-activated Parkinson’s paradigm. Methods: To evaluate neuroprotective activity, rotenone (1.5 mg/kg s.c) and europinidin (10 mg/kg and 20 mg/kg) was administered in rats for 21 days. The behavioural parameters were performed before sacrificing the rats. On the 22nd day, all the rats were assessed for biochemical markers (SOD, GSH, MDA, Catalase), neurotransmitter levels (Dopamine, 5-HIAA, DOPAC, and HVA levels), and neuroinflammatory markers (IL-6, IL-1β and TNF-α). Results: It was found that rotenone produced significant (*p* < 0.001) oxidative damage, a cholinergic deficit, dopaminergic loss, and a rise in neuroinflammatory markers in rats. Conclusion: The study concludes that europinidin possesses anti-oxidant and anti-inflammatory properties. The results suggest the therapeutic role of europinidin against rotenone-activated behavioural, biochemical, and neuroinflammatory alterations in rats.

## 1. Introduction

Parkinson’s disease (PD) is a progressive neuroinflammatory condition triggered by various factors, mainly genetics, mitochondrial imbalance, oxidative stress, and environmental factors. The characteristic motor symptoms are rigidity, akinesia, bradykinesia, and tremor, which occur due to loss in the dopaminergic neurons depleting the dopamine levels in the nigrostriatal area [1,2]. Usually, patients suffering from PD develop Lewy bodies which are formed by α-synuclein agglomeration, resulting in cognitive deficits [3]. The cause of dopaminergic loss is oxidative stress generated in the brain. The reactive free radical produced by oxidative damage cause mitochondrial dysfunction resulting in protein aggregation and eventually cell injury and apoptosis [4,5].

Rotenone is an organic heteropentacyclic compound obtained from the Lonchocarpus species which is used as an insecticide and pesticide. Rotenone when administered inhibits the electron transport chain (ETC) system by binding with mitochondrial complex I [6]. It is the complex-I blocker that has been stated as toxic to dopaminergic neurons both in vivo and in vitro [7]. It can penetrate the blood–brain barrier because of its lipophilic structure and cause aggregation of alpha-synuclein in the brain [8]. Sporadic PD (non-familial) is mainly caused due to depletion in complex I activity. Rotenone produces pathological symptoms of PD including loss of dopaminergic neurons, rigidity, postural imbalance, and behavioral deficits when exposed to systemic circulation [9]. Therefore, rotenone is a unique inducing animal model as it mimics the early symptoms of Parkinsonism with that in humans [10]. Long-term use of levodopa may cause motor fluctuations and dyskinesia, but it is the most effective treatment for PD. A limited number of treatment options are available for PD, most of which involve restoring the dopaminergic tone in the striatum. When administered to rodents, levodopa does not provide the same level of neuroprotection [11].

Europinidin, an o-methylated derivative of delphinidin obtained from the plant *Plumbago Europea* and *Ceratostigma plumbaginoides* belongs to the family Plumbaginaceae [12]. Plumbago Europea is a perineal herb that has wide medicinal applications in treating various disease conditions such as cancer, immunosuppressant, dysmenorrhea, hepatitis, respiratory disorders, edema, and scabies [13,14,15]. Europinidin, an active phytochemical, has not been widely studied for its therapeutic applications, and little literature is available. Moreover, no evidence indicated the influence of europinidin against rotenone-induced PD in rodents. As far as we know, this is the first research study that investigates the effectiveness of europinidin as a therapeutic agent for rotenone-induced Parkinson’s disorder. Hence, the scope of the study is to explore the effect of europinidin in the rotenone-activated Parkinson’s model in rats.

## 2. Results

### 2.1. Acute Toxicity Study

Europinidin was considered a safe oral dose. While performing an acute toxicity study, no mortality or clinical symptoms were observed for 14 days. Therefore, based on acute oral toxicity studies data, we chose 10 and 20 mg/kg Europinidin for the main study. 

### 2.2. Behavioural Studies

#### 2.2.1. Catalepsy Test

**Figure 1:**
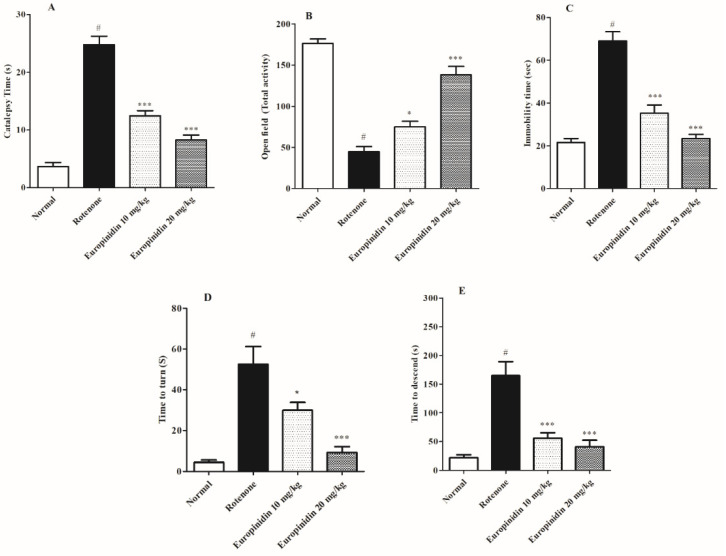
Effect of europinidin on behavioural parameters. (**A**) Catalepsy time, (**B**) Open field activity, (**C**) Immobility time, (**D**) t-turn, (**E**) t-total. The data were expressed as mean ± S.E.M. (*n* = 6/group). * *p* < 0.05, *** *p* < 0.001 when compared with control group; # *p* < 0.001 when compared with rotenone group (one-way ANOVA followed by Tukey’s test).

The rotenone-induced group markedly raised (*p* < 0.001) the catalepsy time when collated to the controls. The europinidin-treated groups at both doses (10 mg/kg and 20 mg/kg) reduced (*p* < 0.001) the catalepsy time when correlated to the rotenone-induced group (Figure 1A).

#### 2.2.2. Open-Field Test

Animals injected with rotenone showed a sharp decline (*p* < 0.001) in the overall test activity correlated to the normal group. The europinidin-treated groups at both doses (10 mg/kg and 20 mg/kg) improved (*p* < 0.05 and *p* < 0.001) the total activity measured when collated to the rotenone-treated group (Figure 1B).

#### 2.2.3. Forced-Swim Test

The rotenone-induced group remarkably raised (*p* < 0.001) the immobility time when collated with the controls, whereas the treatment group at both doses (10 mg/kg and 20 mg/kg) notably decreased (*p* < 0.001) the immobility time when correlated to the rotenone-induced group (Figure 1C).

#### 2.2.4. Pole Test

Rotenone-treated rats (group II) indicated a marked rise (*p* < 0.001) in the t-turn and t-total activity compared to the normal group (Figure 1D,E). Treatment with europinidin (10 and 20 mg/kg) showed lowered (*p* < 0.001) measurements (t-turn and t-total) as compared to the rotenone-induced group except for 10 mg/kg europinidin, which showed lowered t-turn time (*p* < 0.05).

### 2.3. Biochemical Analysis

#### 2.3.1. Oxidative Stress Parameters

**Figure 2:**
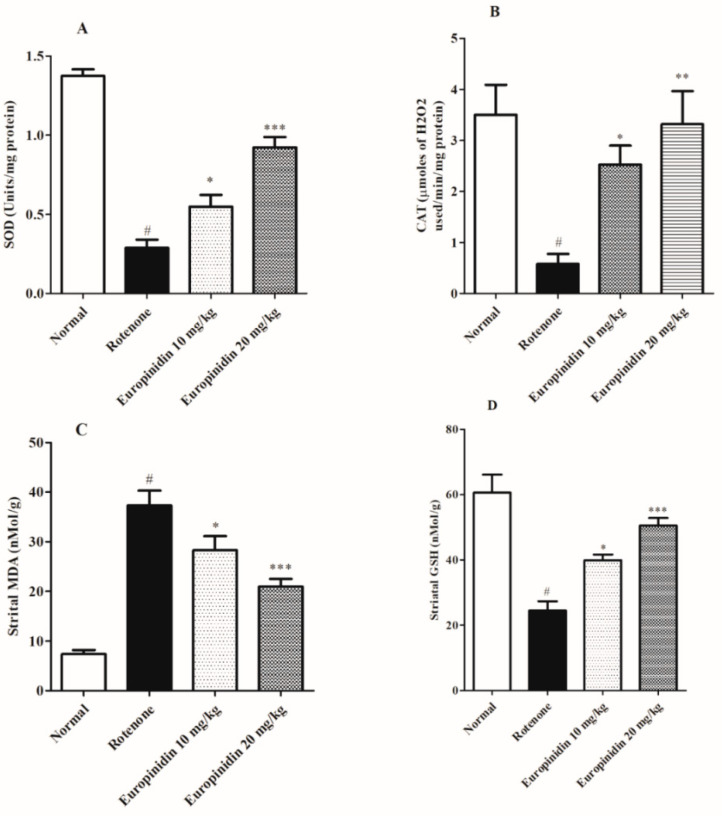
Effect of europinidin on antioxidant enzymes. (**A**) Superoxide dismutase (SOD), (**B**) Catalase (CAT), (**C**) Malondialdehyde (MDA), (**D**) Glutathione transferase (GSH). The data were expressed as mean ± S.E.M. (*n* = 6/group). * *p* < 0.05, ** *p* < 0.01, *** *p* < 0.001 when compared with the control group; # *p* < 0.001 when compared with rotenone group (one-way ANOVA followed by Tukey’s test).

The rotenone-induced group showed remarkably low (*p* < 0.001) levels of GSH, SOD, and CAT and increased the MDA level when collated to the controls. Treatment with europinidin at both doses (10 mg/kg and 20 mg/kg) significantly elevated (*p* < 0.05 and *p* < 0.001) the level of SOD and GSH, while theCAT level was also raised (*p* < 0.05 and *p* < 0.01) markedly at both doses (10 and 20 mg/kg) as collated to the rotenone group. The rotenone-induced group remarkably raised (*p* < 0.001) the MDA level when collated to controls. The europinidin-treated group at both doses lowered (*p* < 0.05 and *p* < 0.001) the MDA level when correlated to the rotenone-induced group (Figure 2A–D).

#### 2.3.2. Acetylcholinesterase Activity (AChE)

**Figure 3:**
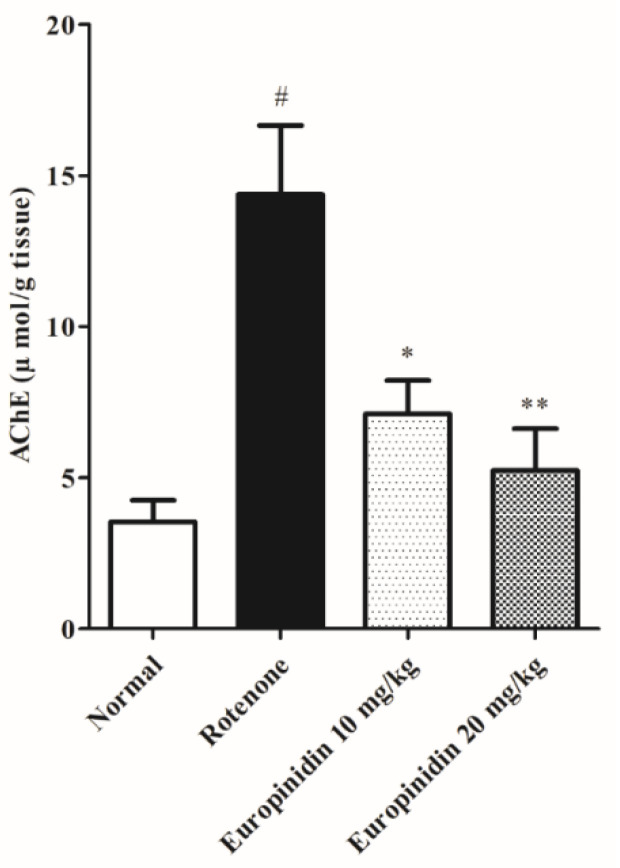
Effect of europinidin on AChE. The data were expressed as mean ± S.E.M. (*n* = 6/group). * *p* < 0.05, ** *p* < 0.01, when compared with the control group; # *p* < 0.001 when compared with the rotenone group (one-way ANOVA followed by Tukey’s test).

Rotenone administration remarkably increases (*p* < 0.001) the AChE level when collated to the normal. Treatment with europinidin at both doses (10 and 20 mg/kg) showed a significant decline (*p* < 0.05 and *p* < 0.01) in AChE activity when collated with the rotenone group (Figure 3).

### 2.4. Effect of Europinidin on Neurotransmitter Levels

**Figure 4:**
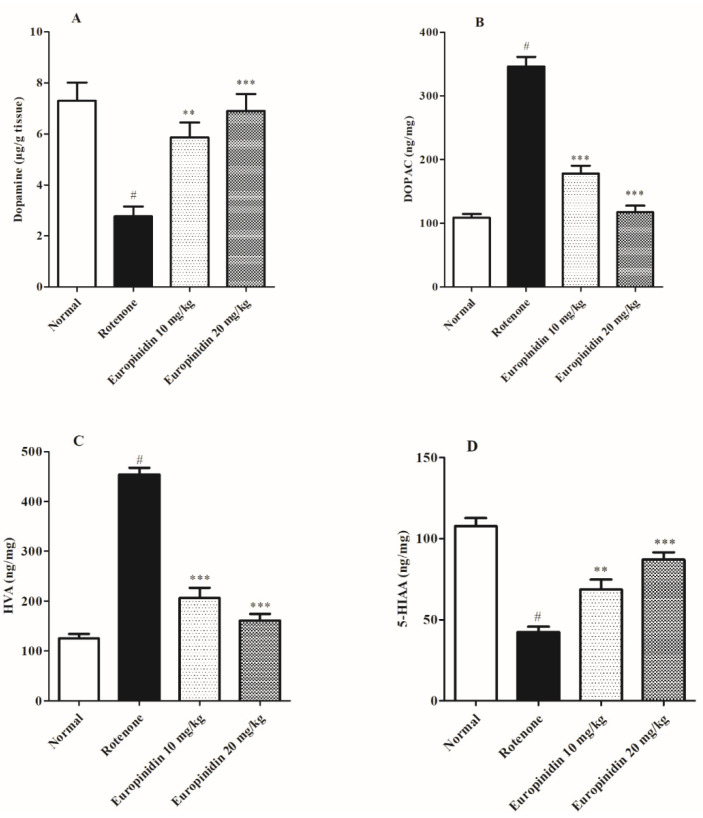
Effect of europinidin on neurotransmitter levels. (**A**) Dopamine, (**B**) DOPAC, (**C**) HVA concentrations, (**D**) 5-HIAA concentrations. The data were expressed as mean ± S.E.M. (*n* = 6/group). ** *p* < 0.01, *** *p* < 0.001 when compared with the control group; # *p* < 0.001 when compared with the rotenone group (one-way ANOVA followed by Tukey’s test).

Rotenone administration revealed a pronounced decrease (*p* < 0.001) in dopamine and 5-HIAA and a rise in HVA and DOPAC levels when collated to normal animals. The europinidin-treated group at both doses elevated (*p* < 0.01 and *p* < 0.001) the levels of dopamine and 5-HIAA, while a considerable decline (*p* < 0.001) in the level of DOPAC and HVA was noted when collated to the rotenone group (Figure 4A–D).

### 2.5. Effect of Europinidin on Neuroinflammatory Markers

**Figure 5:**
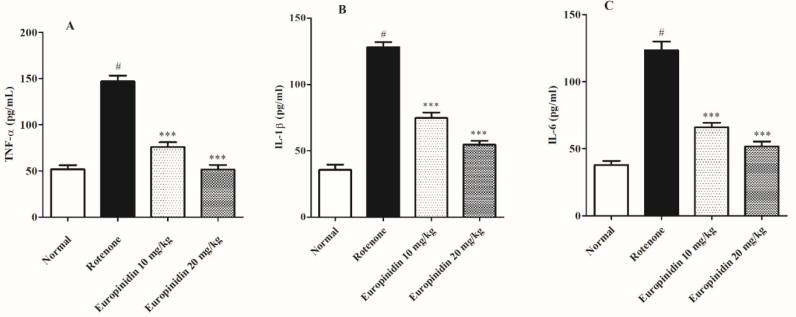
Effect of europinidin on neuroinflammatory parameters. (**A**) TNF-Alpha, (**B**) IL-1ß, (**C**) IL-6. The data were expressed as mean ± S.E.M. (*n* = 6/group). *** *p* < 0.001 when compared with the control group; # *p* < 0.001 when compared with rotenone group (one-way ANOVA followed by Tukey’s test).

Administration of rotenone markedly increased (*p* < 0.001) the IL-1β, TNF-α, and IL-6 levels when collated to the normal group. The europinidin-treated group at both doses decreases (*p* < 0.001) IL-1β, IL-6, and TNF-α levels as collated to the rotenone group (Figure 5A–C).

## 3. Materials and Methods

### 3.1. Chemicals and Reagents

Europinidin, rotenone (Sigma-Aldrich), chemicals (Modern Lab, Maharashtra, India), and kits for tumor necrosis factor-alpha (TNF-α), interleukin-6 (IL-6), and IL-1β were analyzed by a rat enzyme-linked immunosorbent assay kit (Sigma-Aldrich, Louis, MO, USA) and used to quantity.

### 3.2. Animals

Male Wistar rats (150–180 g) were acclimatized for a week. The rats were grouped and stored in propylene cages at standard room temperature (24 ± 5 °C) with relative humidity (50–65%) under a 12–12 h light/dark cycle. Pellet food and free access to water were provided to rats. The study was passed by the institutional ethics committee for animals (IAEC/918/CPCSEA/01) and carried out following CPCSEA guidelines.

### 3.3. Experimental Plan

A total of 24 rats were segregated into 4 groups, each group containing 6 rats.

Cluster I: Normal group received sunflower oil (s.c).

Cluster II: Rotenone −1.5 mg/kg s.c.

Cluster III-IV: Rotenone + Europinidin −10 and 20 mg/kg/p.o.

**Figure 6:**
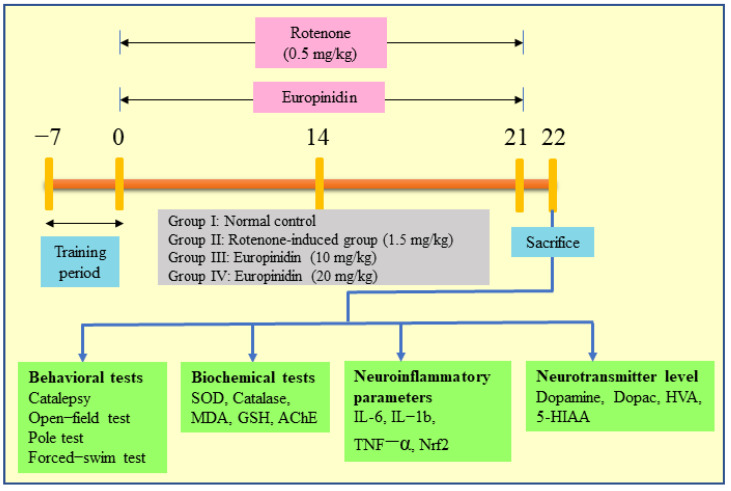
Brief presentation of the experimental protocol.

Rotenone was subcutaneously dosed (1.5 mg/kg) for 21 days [16,17]. Treatment groups received Europinidin in both doses for 21 days, 1 h before rotenone injection (Figure 6). The rats were subjected to behavioral tests after 24 h of the last rotenone injection and then immediately euthanized under light anesthesia. The brains were collected and cleaned with cold saline and stored in formalin solution at 4 °C. 

### 3.4. Acute Toxicity Study

The study followed OECD guideline no. 423. All the animals were examined for 14 days for sign of toxicity. The rats were also examined for any clinical signs, including changes in behaviour and body weight [18]. Europinidin was given orally in rats as per the previously published toxicity studies [19,20,21].

### 3.5. Behavioural Assessment

#### 3.5.1. Catalepsy Test

The Catalepsy/Bar test was performed by placing the horizontal bar 9 cm above the surface. The paws were kept on the bar by holding and lowering the forepaws until the tail reached the ground. The time at which the front paws touched the ground was noted by a stopwatch. The sessions were video-recorded for evaluating the time and comparing the performance of each rat. The cut-off time for each rat was fixed to 3 min [22,23].

#### 3.5.2. Open-Field Test

The test contains a rectangular open field with a floor covered with cloth which was divided into 25 squares (20 × 20 cm). The rats were kept in the middle square and observed for locomotor activity. The distance displaced by the rat, no rearing, time taken at the initial position, and the number of entries in the central square was noted. The overall activity of the test was calculated [24,25].

#### 3.5.3. Pole Test

The test is generally used in rodents to determine disorientation in movement. It consists of a vertical wooden pole (50 cm) with its base confined in a home cage. The rats were kept on the pole and the time to descend the pole was noted. The rats were given training sessions for 2 days prior to starting the test. On the test day, five trials were performed and the mean of trials was used for calculation. The time taken by each rat to align downward (time to turn) and the time to descend were noted [26,27].

#### 3.5.4. Forced-Swim Test

The test consists of a cylindrical tank filled with water with a depth of at least 35 cm. The rats were trained prior to performing the main test by placing the rats in the water-filled tank. On the next day, the test session was done by placing the rats in the tank for 5 min. The test was video-recorded to assess the following parameters: immobility time (i.e., no body movements), swimming (i.e., motions by forepaws that displaced water), and climbing (i.e., vigorous movement to force out from the side of the tank wall). The water was changed and cleaned during every session [28,29].

### 3.6. Biochemical Analysis

#### 3.6.1. Homogenization of Brain

On the 22nd day, after a behavioural assessment, the animals were separated for estimation of biochemical, neurotransmitter levels, and neuroinflammatory markers. The collected brains were homogenized using phosphate buffer. The supernatant was obtained by centrifugating the homogenate at 15,000–25,000 rpm for 25 min. The resultant mixture was used for further analysis.

#### 3.6.2. Malondialdehyde (MDA) Estimation

MDA is the end product that describes the extent of lipid peroxidation. The centrifuge tube contains homogenate, acetic acid (20%), dodecyl sulfate (8%), and thiobarbituric acid (0.8%). The reaction tube was boiled at 90 °C for 60 min, cooled, and centrifugated at 1500–2000 rpm for 15 min. The mixture was estimated spectrometrically at 532 nm. The amount of MDA produced was estimated and expressed as nmol/g [30,31].

#### 3.6.3. Reduced Glutathione (GSH)

GSH was evaluated by the following process described by Ellman et al. [32]. In the reaction tube, homogenate (0.1 mL), trichloroacetic acid (10%), and Ellman’s reagent (5, 5–0-dithiobisnitro benzoic acid in sodium citrate and phosphate buffer) was mixed. The mixture was centrifugated at 2000 rpm for 15 min. The absorbance was recorded at 412 nm by a UV spectrophotometer. The results were presented as nmol/GSH/g of tissue [33].

#### 3.6.4. Superoxide Dismutase (SOD)

The assay principle works on the reduction of nitrobluetetrazolium (NBT) by the superoxide dismutase. To the supernatant, xanthine oxidase was added to the mixture of nitrobluetetrazolium (NBT) and phosphate buffer in the reaction tube and was incubated. The SOD was presented as units/mg protein, and 1 unit of SOD activity is calculated by the amount of protein that inhibited 50% NBT reduction [34,35].

#### 3.6.5. Catalase Activity (CAT)

To 0.5 mL supernatant, 2 mL phosphate buffer and 1 mL hydrogen peroxide (H_2_O_2_) were added to the cuvette. The absorbance was estimated spectrophotometrically at 240 nm for 30 s. The activity was presented as mmoles of H_2_O_2_ oxidized per minute per milligram protein [36,37]. 

#### 3.6.6. Estimation of Acetylcholinesterase Activity (AChE)

It is an indirect method for the determination of acetylcholine in tissues. To 0.5 mL homogenate, 0.2 M phosphate buffer, and dithiobis- nitrobenzoic acid (DTNB) were mixed to the reaction tube. The absorbance of the reaction mixture was recorded spectroscopically at 412 nm. After a few mins, acetylthiocholine was mixed and the change in the absorbance was noted [38,39].

#### 3.6.7. Neurotransmitter Levels

The neurotransmitters include dopamine (DA), 3,4-dihydroxyphenylacetic acid (DOPAC), homovanillic acid (HVA), and 5-hydroxyindoleacetic acid (5-HIAA), which were estimated by an enzyme-linked immunosorbent assay (Rat ELISA) kits following standard assay procedure.

#### 3.6.8. Neuroinflammatory Markers

The cytokines such as IL-6, IL-1β, and TNF-α were determined by respective ELISA kits. The protein from the homogenate was separated and pipetted in an antibody-coated ELISA plate. The level of cytokines was measured by following the standard assay procedure (Ray Bioassay kit). 

### 3.7. Statistical Analysis

All the statistical values were presented as mean ± SEM. The data were estimated by one-way ANOVA followed by Tukey’s test using GraphPad prism 5.0. One-way ANOVA was used for estimating the significance among the two groups by setting the criteria at *p* < 0.05.

## 4. Discussion

Europinidin, an antioxidant, scavenges the reactive oxygen species generated in the mitochondria which may decrease oxidative damage [40]. The current investigation for the first time proved that europinidin ameliorated rotenone-induced Parkinson’s disease in animals at 10 mg/kg and 20 mg/kg dose levels. Previous findings show that the rotenone induction model reproduces behavioral, inflammatory, and biochemical alterations in rats which are similar to PD symptoms [41,42,43]. Rotenone passes through the BBB and modifies the neurological, behavioral, and oxidative pathways of PD [44]. Rotenone administration impaired the motor performance and cognitive functions as displayed in behavioral tests, i.e., catalepsy, pole test, open-field test, and the Morris water test, which were in line with previous investigations [45,46]. Treatment with europinidin dose-dependently improved the learning ability and corrected cognitive deficits, proving the role of europinidin in modulating the deficit signaling pathway.

Several reports have emphasized the effect of oxidative stress as a crucial factor in deteriorating dopaminergic neurons [47]. The results showed that administration of rotenone ameliorated oxidative damage by decreasing SOD, GSH, and catalase activity and elevating MDA levels in the rat striatum, which were in line with the earlier research studies [48,49]. Treatment of europinidin at both doses (10 mg/kg and 20 mg/kg) significantly restored the antioxidant level by decreasing the oxidative damage produced in the neurons.

Various research findings showed that the cholinergic system plays a key role in regulating neurological functions such as learning, motor performance, memory, and sleep [50,51]. Acetylcholinesterase, a specific enzyme marker mainly located in postsynaptic synapses, hydrolyzes acetylcholine (Ach) into acetic acid and choline. In line with earlier reports, the current study results also showed that rotenone administration markedly increases AChE activity [52,53]. A concomitant decline in AChE activity leads to a rise in the synaptic acetylcholine level that is required to modulate cognitive performance. Our study suggests that rats treated with europinidin dose-dependently reduced AChE activity, thereby minimizing the hydrolyzation and conversion of acetylcholine so that a sufficient amount of Ach could be available in the synaptic cleft.

Oxidative damage is the major cause of the depletion of neurotransmitters, mainly dopamine, which essentially plays a role in controlling functional movements and signals transmission [54]. In the present study, rotenone significantly decreased dopamine levels and also altered the concentrations of its metabolites, which were similar to previous findings [55,56]. Treatment with europinidin dose-dependently restored the levels of neurotransmitters near to normal, indicating improvement in the neuronal and behavioural functions and the anti-oxidant role of europinidin in PD.

Several reports have shown that inflammation has a major role in the progression of PD. The activation of the neuroinflammatory pathway triggers the expression of the cytokines including IL-1b, TNF-α, and IL-6 in the microglia [57]. In line with earlier findings, the present study showed a sharp rise in inflammatory cytokine levels in the rotenone- activated group [58,59]. Treating with europinidin at both doses displayed a significant decline in the cytokine level, which proved that europinidin possesses anti-inflammatory properties. Europinidin antioxidant qualities may be responsible for its protective benefits in rats suffering from rotenone-induced neurotoxicity. These findings also suggest that europinidin may also contribute to the anti-inflammatory property by preventing oxidative stress and cytokine levels, improving cholinergic function, and up-regulating DA release and mitochondrial deficits in the brain. As a result, europinidin antioxidant therapy attenuates oxidative damage underlying mitochondrial dysfunction and may be beneficial in treating PD. We will be performing future molecular-level studies to confirm this mechanism.

## 5. Conclusions

The study revealed that the neuroprotective effect of europinidin in rotenone-activated Parkinson’s disease was due to its ability to overturn cognitive deficits and behavioural patterns, alter the oxidative injury by restoring the antioxidant enzymes, decreasing the neuroinflammation, and recovering the neurotransmitter level. Hence, europinidin could be a good candidate and can be used as a therapeutic agent in treating PD. Further studies should be done on finding the molecular mechanism of europinidin associated with neuroprotection by considering higher dose levels. Further europinidin at a higher dose, i.e., 20 mg/kg showed greater protection against rotenone activated dysfunctions.

## Data Availability

All the data given in the manuscript.
